# Can I solve my structure by SAD phasing? Planning an experiment, scaling data and evaluating the useful anomalous correlation and anomalous signal

**DOI:** 10.1107/S2059798315019403

**Published:** 2016-03-01

**Authors:** Thomas C. Terwilliger, Gábor Bunkóczi, Li-Wei Hung, Peter H. Zwart, Janet L. Smith, David L. Akey, Paul D. Adams

**Affiliations:** a Bioscience Division, Los Alamos National Laboratory, Mail Stop M888, Los Alamos, NM 87545, USA; b Department of Haematology, University of Cambridge, Cambridge Institute for Medical Research, Wellcome Trust/MRC Building, Cambridge CB2 0XY, England; c Physics Division, Los Alamos National Laboratory, Mail Stop D454, Los Alamos, NM 87545, USA; d Physical Biosciences Division, Lawrence Berkeley National Laboratory, Berkeley, CA 94720, USA; e Life Sciences Institute, University of Michigan, Ann Arbor, MI 48109, USA; f Department of Biological Chemistry, University of Michigan, Ann Arbor, MI 48109, USA

**Keywords:** SAD phasing, anomalous signal, experimental design

## Abstract

A key challenge in the SAD phasing method is solving a structure when the anomalous signal-to-noise ratio is low. Here, algorithms and tools for evaluating and optimizing the useful anomalous correlation and the anomalous signal in a SAD experiment are described. A simple theoretical framework [Terwilliger *et al.* (2016 ▸), *Acta Cryst.* D**72**, 346–358] is used to develop methods for planning a SAD experiment, scaling SAD data sets and estimating the useful anomalous correlation and anomalous signal in a SAD data set. The *phenix.plan_sad_experiment* tool uses a database of solved and unsolved SAD data sets and the expected characteristics of a SAD data set to estimate the probability that the anomalous substructure will be found in the SAD experiment and the expected map quality that would be obtained if the substructure were found. The *phenix.scale_and_merge* tool scales unmerged SAD data from one or more crystals using local scaling and optimizes the anomalous signal by identifying the systematic differences among data sets, and the *phenix.anomalous_signal* tool estimates the useful anomalous correlation and anomalous signal after collecting SAD data and estimates the probability that the data set can be solved and the likely figure of merit of phasing.

## Abstract 2

Algorithms for evaluating and optimizing the useful anomalous correlation and the anomalous signal in a SAD data set are described.

## 1. Introduction   

The single-wavelength anomalous diffraction (SAD) method is a widely used experimental phasing technique (Dauter *et al.*, 2002 ▸; Hendrickson, 2014 ▸) that accounts for over 70% of depositions of experimentally phased structures in the Protein Data Bank (PDB; Berman *et al.*, 2000 ▸). It exploits differences observed between the intensities of reflections that are related by inversion symmetry (Bijvoet, 1954 ▸) caused by the element- and wavelength-dependent X-ray absorption characteristics of atomic scatterers. Although this effect is universally present in the Sohncke space groups most commonly encountered in macromolecular crystallography, it is often not detectable because of its small magnitude. The use of anomalous differences in experimental phasing typically requires a specific element to be present at a limited number of sites in the macromolecular structure and the choice of an X-ray wavelength near an absorption edge. The ‘anomalous’ differences are used to locate the atoms in the substructure (Weeks *et al.*, 1993 ▸; Terwilliger & Berendzen, 1999 ▸; Schneider & Sheldrick, 2002 ▸; Grosse-Kunstleve & Adams, 2003 ▸) and are then used along with the substructure to estimate phases for the entire structure (Otwinowski, 1991 ▸; de La Fortelle & Bricogne, 1997 ▸; Furey & Swaminathan, 1997 ▸; McCoy *et al.*, 2004 ▸; Pannu & Read, 2004 ▸).

In this work, we use the anomalous signal, defined as the peak height in a model-phased anomalous difference Fourier map at coordinates of atoms in the anomalous substructure (Yang *et al.*, 2003 ▸), as a measure of the information about the substructure present in an anomalous data set. It has been shown in Terwilliger *et al.* (2016 ▸) that the anomalous signal is a good predictor of the ability to locate atoms in the substructure and that it is related to the experimental data by a simple relationship,
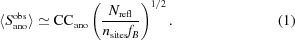
Here, *S*_ano_^obs^ denotes the observed anomalous signal and CC_ano_ is the useful anomalous correlation (Terwilliger *et al.*, 2016 ▸). The ‘useful anomalous correlation’ is defined as the correlation of observed anomalous differences with ideal anomalous differences from the final refined structure but considering anomalous scattering only from the *n*_sites_ atom included in the anomalous substructure. It is essentially a measure of how similar the observed anomalous differences are to the best set of anomalous differences that could possibly be obtained for the non-anomalously scattering atoms of this crystal and its detectable anomalous substructure. All of the quantities in (1) refer to the data set as a whole, so that, for example, CC_ano_ is the overall mean useful anomalous correlation. The resolution-dependence of measurements of anomalous differences is reflected in CC_ano_, which accordingly will typically become lower as high-resolution data are included. The number of unique reflections in the data set is *N*_refl_. The factor *f_B_* is the second moment of the scattering factors of the anomalous substructure, calculated using the anomalous scattering factors *f*_*h*,*B*_ (including atomic displacement) at the resolution corresponding to each reflection in the data set,
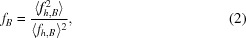
where the average is over all reflections in the data set. The factor *f_B_* is also dependent on resolution, typically becoming larger when higher resolution data are included. The combined effect of the decrease in useful anomalous correlation CC_ano_ and the increase in *f_B_* with the addition of high-resolution data is that the overall anomalous signal does not normally increase proportionally to the square root of the number of reflections and can even decrease (if random anomalous differences are added).

In this work, we describe methods for scaling and merging anomalous data from multiple crystals or data collections and for estimating the useful anomalous correlation CC_ano_ and the anomalous signal *S*_ano_^obs^ at different stages of the experiment. We also develop empirical relationships between the anomalous signal *S*_ano_^obs^ and the probability of solving the substructure, and between the useful anomalous correlation CC_ano_ and the expected quality of initial phases.

## 2. Methods   

### 2.1. Scaling and merging anomalous data   

Our procedure for scaling and merging SAD data from any number of crystals or orientations of crystals involves seven steps that are an expanded version of the local scaling procedure used previously in *SOLVE* (Terwilliger & Berendzen, 1999 ▸) and *phenix.autosol* (Terwilliger *et al.*, 2009 ▸). These are the following.

(i) Grouping of data sets with similar unit-cell parameters and choice of the largest group, removing all other data sets from consideration.
(ii) Calculation of and correction for the average anisotropy of all of the data sets under consideration.
(iii) Splitting of data sets into sub-data sets containing at most one observation of each unique index.
(iv) Local scaling of each individual sub-data set.
(v) Scaling of each sub-data set to a merged data set with an overall scale factor.
(vi) Estimation of systematic differences between each individual sub-data set and a merged data set.
(vii) Merging and averaging the scaled sub-data sets, including the estimates of systematic differences between individual data sets and the merged data set in the weighting of each measurement.

These steps are described in groups in the following sections.

#### 2.1.1. Choice of data sets and correction to averaged anisotropy   

The first steps in our procedure for scaling and merging of SAD data sets includes the choice of which data sets to include in the process and the application of an anisotropy correction to each data set so that all of the data sets have anisotropy similar to the average of all of the original data sets. When data sets from several crystals are available, a group of data sets from crystals with similar unit-cell parameters is chosen. The required similarity among data sets can be adjusted by the user. The default criteria for similarity are unit-cell lengths matching within 1% and unit-cell angles matching within 1°. An alternative criterion available for unit-cell lengths is that they must match within a specified fraction (typically one quarter to one half) of the high-resolution limit of the data (Drenth, 1999 ▸). The group of data sets with the largest total number of measured reflections satisfying these criteria are chosen and the remainder of the data sets are set aside.

Next, the average anisotropy parameters for the remaining data sets are estimated using the tools in *phenix.xtriage* (Zwart, 2005 ▸; Zwart *et al.*, 2005 ▸). The anisotropy parameters for all of these data sets are averaged to estimate the average anisotropy. The data in all data sets are then adjusted so that each data set has this average anisotropy. In this way, differences among the data sets are minimized while maintaining the anisotropy and fall-off with resolution of the data sets as a whole.

Once the data sets have been selected and brought to a common anisotropy, they are split, if necessary, into smaller data sets containing at most one observation corresponding to each unique index (*h*, *k*, *l*), where in this context ‘unique’ refers to the entire limiting sphere, not the reciprocal asymmetric unit. This step is included so that the subsequent local scaling step can be carried out on data sets that contain no duplicate measurements. The assumption is made that unmerged datafiles will group symmetry-related reflections together, and within such a group of reflections the reflections will be ordered based on order of measurement. The splitting of measurements into separate sub-datafiles is therefore carried out considering reflections in the order that they are present in the input datafile. Within a group of symmetry-related reflections, for any reflection for which the indices duplicate a set of indices already found, the corresponding reflection is placed in a new sub-datafile. When a datafile is split into sub-datafiles, any sub-datafiles that contain less than 30% of the number of reflections in the largest sub-datafile are typically discarded. Additionally, in order to reduce systematic errors, an attempt is made to match Bijvoet mates within each data set and to exclude measurements of acentric reflections for which no Bijvoet mate was measured.

#### 2.1.2. Local scaling of each individual data set or sub-data set   

The key scaling algorithm applied to an individual data set or sub-data set in our procedure is local scaling (Matthews & Czerwinski, 1975 ▸) as implemented in *SOLVE* and *phenix.autosol* (Terwilliger & Berendzen, 1999 ▸; Terwilliger *et al.*, 2009 ▸; Adams *et al.*, 2010 ▸). The idea of local scaling is that any systematic errors that affect the intensity of a particular reflection will tend to similarly affect the intensities of reflections nearby in reciprocal space, which were presumably recorded at nearly the same time during data collection. If data set *A* is to be scaled to data set *B*, a local scale factor can be calculated for each reflection in data set *A* based on the ratio of the intensities of nearby reflections in data set *B* to the corresponding reflections in data set *A*. The reflections for calculating the scale factor are chosen in pairs symmetrically arranged around the reflection to be scaled if possible, and typically 30 or more reflections are chosen for estimating the scale factor. The scaling procedure is carried out using amplitudes (Terwilliger & Berendzen, 1999 ▸), but we emphasize that the input data are unmerged intensities with original *hkl* indices as assigned by the indexing and integration software. The intensities are converted to amplitudes by taking their square roots (or setting them to zero if negative) and they are converted back to intensities at the end of the procedures by squaring.

The procedure carried out here for local scaling of a single data set begins by averaging all observations of each unique reflection in the reciprocal-space asymmetric unit to create an average data set to be used as a scaling target. The original unmerged data set is then split into two: one with all the (*F* ^+^) observations that are symmetry-equivalent to each reflection in the reciprocal-space asymmetric unit of the crystal and one with the Bijvoet mates (*F* ^−^) of these reflections. Each of these partial data sets is scaled to the average data set with local scaling. The *F* ^+^ and *F* ^−^ data sets are then combined without further scaling to create a scaled anomalous data set with scaled *F* ^+^ and *F* ^−^ values for each reflection in the reciprocal-space asymmetric unit.

#### 2.1.3. Estimation of systematic errors or features unique to each data set and merging including estimates of systematic differences   

As the data sets that are to be merged may come from different crystals and after different exposures of an individual crystal, the anomalous differences for the various data sets, even if measured and scaled perfectly, might not all be equal. Our algorithm for scaling multiple data sets attempts to identify the intrinsic differences between each individual data set and the mean (or a selected data set) and then weights the anomalous differences from each data set including these data-set-based intrinsic differences. The procedure is first to convert each pair of measurements of *F* ^+^ and *F* ^−^ to estimates of the mean structure-factor amplitude 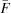 and the anomalous difference Δ_*i*_. Next, an average data set is created by simple merging of all of the observations of a particular anomalous difference Δ_*i*_ from all data sets *i*, with weights *w_i_* for each observation based on the experimental uncertainties,
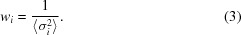
Estimates of the intrinsic differences between anomalous differences in each data set and the average (*D_i_* ^2^) are then obtained by subtracting the sum of mean-square error estimates for the mean (〈σ_m_^2^〉) and individual data sets (〈σ_*i*_^2^〉) from the observed mean-square difference between the average and individual data sets [〈(Δ_*i*_ − Δ_m_)^2^〉],
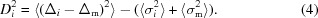
The logic of (4) can perhaps be most easily understood by considering the case where many data sets are collected so that Δ_m_ is essentially the true mean anomalous difference for an average crystal. In this case, the mean-square difference between an individual measurement Δ_*i*_ and the true anomalous difference for an average crystal Δ_m_ would be the sum of the mean-square measurement error in Δ_*i*_ (〈σ_*i*_^2^〉) and the mean-square difference between anomalous differences for crystal *i* and the average crystal (*D_i_* ^2^). This calculation slightly underestimates the intrinsic differences because the mean anomalous difference (Δ_m_) includes the value from the individual data set (Δ_*i*_); however, if there are many data sets included the effect will be small and it is ignored here. Finally, the anomalous differences from all data sets are again averaged together, this time including the intrinsic differences in the weights,
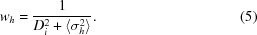
The resulting anomalous differences are then recombined with the estimates of the average structure factor (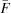) to yield new estimates of the members of the Bijvoet pair *F* ^+^ and *F* ^−^. 

### 2.2. Calculation of metrics of anomalous data quality   

We calculated the values of three metrics of anomalous data quality. One is based on the half-data-set anomalous correlation, one on the skew of the anomalous difference Patterson and one on estimates of the ratio of the mean-square errors in measurement to the mean-square anomalous differences. These metrics of data quality will form the basis for Bayesian estimation of the useful anomalous correlation CC_ano_ for a data set.

#### 2.2.1. Calculation of half-data-set anomalous correlation   

A half-data-set anomalous correlation is generally obtained (Evans, 2006 ▸) by randomly assigning each reflection in a data set into one of two groups, merging the data within each group and calculating the correlation of anomalous differences between the groups. In this work, several data sets were typically combined to create merged data sets and we anticipated that the Bijvoet pairs in one data set might be systematically different from those in another data set. We attempted to minimize the effects of such systematic differences by keeping reflections measured from one crystal together when the half data sets are created. If multiple SAD data sets were available for a particular crystal structure, the data sets were divided into two approximately equally sized groups, each half data set was scaled with local scaling as described above and the correlation of anomalous differences between the two half data sets was calculated. If only a single unmerged data set was available, it was split by choosing approximately half of the observations of each unique reflection to be in each of two half data sets. If the observations for a unique reflection were grouped together in the data file, the reflections were simply split into approximately the first and second half as they appeared in the file, otherwise they were chosen randomly.

Using the half-data-set anomalous correlation we calculated a metric that we anticipated would be a useful predictor of the useful anomalous correlation CC_ano_ between the anomalous differences for the entire data set and the anomalous differences expected from the true anomalous substructure. Our metric is related to the widely used estimator for the expected correlation of the intensities in a merged data set with true intensities (CC*; Karplus & Diederichs, 2012 ▸). If a data set containing multiple measurements of intensities for each reflection is divided into two parts, each containing half of the measurements of each reflection, then the expected correlation CC* of the intensities in the merged data set with the true intensities is given approximately by (Karplus & Diederichs, 2012 ▸)
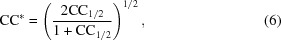
where CC_1/2_ is the correlation between intensities in the two half data sets, known as the half-data-set correlation. A key assumption in deriving this formula is that the observations for each reflection can be split into two equally sized groups. Following the same analysis, substituting the half-data-set anomalous correlation for the half-data-set correlation of intensities, and considering the possibility that only a fraction *w* of the reflections have been measured more than once, the expected correlation of anomalous differences in a merged data set with the true anomalous differences is given approximately by
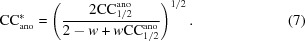
As the value of the estimated anomalous correlation CC* is not defined when the measured half-data-set correlation CC_1/2_ is negative, our metric for anomalous data quality based on the half-data-set correlation is instead the square of the expected correlation, 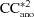,
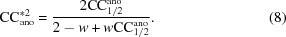
As in (1), the values of these correlations are for the data set as a whole (after scaling and merging). The values of the correlations will normally decrease when higher resolution data are added.

#### 2.2.2. Calculation of the skew of an anomalous difference Patterson function   

An anomalous difference Patterson function normally has strong positive peaks corresponding to vectors between coordinates of atoms in the anomalous sub­structure. A very high peak is always present at the origin. Additionally, high peaks corresponding to translations that are part of noncrystallographic symmetry may also be present (Read *et al.*, 2013 ▸). The skew (the third moment) of a function reflects the positive and negative distributions of values in the function. In the case of an anomalous difference Patterson, the skew is generally positive if there are strong positive peaks and few negative peaks. We have previously used the skew of an electron-density map (Podjarny, 1976 ▸; Lunin, 1993 ▸) as a measure of its quality (Terwilliger *et al.*, 2009 ▸). Here, we use a modified calculation of the skew of the difference Patterson function as a metric of anomalous signal. The procedure is to calculate an origin-removed anomalous difference Patterson map, adjusting the mean to zero and calculating the r.m.s. (σ) of the resulting map, and then to truncate the map at ±4σ. Optionally, the map is further truncated at ±3σ at all points where the native Patterson function has a value outside the range of ±3σ in order to reduce the effect of any translational noncrystallographic symmetry (the default is not to carry out this truncation). The skew *s* of the resulting adjusted anomalous difference Patterson is then calculated,
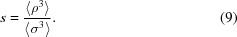
Although there is no simple relationship between the skew of the anomalous difference Fourier and the useful anomalous correlation CC_ano_ between the anomalous differences and those expected from the anomalous substructure, we anticipated that a higher value of CC_ano_ might be associated with higher values of the skewness of the map.

#### 2.2.3. Estimation of errors in measurement of anomalous differences   

During the process of measuring diffraction spots and estimating X-ray intensities, the uncertainties in the intensities are normally estimated as well. It is well known, however, that it is difficult to estimate these uncertainties accurately (Dauter, 2006 ▸; Evans, 2006 ▸). We have adopted a simple procedure for rescaling the uncertainty estimates for a data set. Our procedure is related to the rescaling procedures used in *SCALA* (Evans, 2006 ▸) and in *HKL*-2000 (Otwinowski & Minor, 1997 ▸), but in our procedure the scale factor is obtained using only anomalous differences and using only data from the highest resolution shells. Our procedure is based on two assumptions. One is that the relative uncertainties of measurements within a data set are reasonably accurate, so that an overall scale factor can be applied to all uncertainties to improve their accuracy. The second assumption is that for data measured near the highest resolution obtained in the experiment the anomalous signal is much smaller than the error in measurement. Our procedure for rescaling uncertainties consists simply of finding a scale factor β to apply to the uncertainties that will yield a mean-square uncertainty in anomalous differences in the highest shell of resolution 〈σ^2^_ano_〉 that is equal to the mean-square anomalous difference 〈(Δ^obs^_ano_)^2^〉, 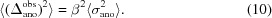
The mean-square values in the highest shell of resolution in this calculation are extrapolated based on the values in several shells of resolution. The tool *get_sigma_ratio* in *phenix.get_patterson_skew* was used to carry out this calculation. All the uncertainties in that data set are then multiplied by the scale factor β. We further calculate a normalized error estimate *e* for the entire data set based on the ratio of the mean-square (rescaled) uncertainties to the mean-square observed anomalous differences,
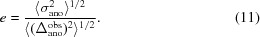
Note that, by construction, for the highest resolution shell the value of *e* is essentially unity. This normalized error estimate *e* was used as a metric representing the overall accuracy with which the anomalous differences were measured. We note that if there were no minor anomalous scatterers in a structure, so that Δ_ano_^other^ = 0, then the overall normalized error in the anomalous differences would be simply equal to *e* and the useful anomalous correlation CC_ano_ would be expected to be (*cf.* Terwilliger *et al.*, 2016 ▸)
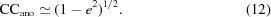



### 2.3. Estimators   

#### 2.3.1. Bayesian estimation of the useful anomalous correlation   

We previously created a simple tool for Bayesian estimation (*bayesian_estimator.py*) and used it to estimate the quality of electron-density maps from features of the map such as its skewness (Terwilliger *et al.*, 2009 ▸). This estimator starts by constructing a two-dimensional histogram from a training set of values of predictor variable/target variable pairs. The estimator then uses the histogram to calculate the variance of the predictor variable for a given value of the target variable. Finally, given a particular value of a predictor variable, it calculates the posterior probability distribution for the target variable using Bayes’ rule and a flat prior probability distribution. When more than one predictor variable is available, the tool assumes independence of the predictor variables and calculates a composite probability for the target variable. Here, we use the same tool to estimate the correlation (CC_ano_) of anomalous differences in a SAD data set to the anomalous differences corresponding to the true substructure. The training data for the Bayesian estimator consists of data from a set of 218 SAD data sets (166 of which have unmerged data available) taken from SAD or MAD data deposited in the PDB for 113 structures (see the list in §[Sec sec2.4]2.4), truncated at resolutions varying from 1.5 to 6 Å to yield a total of 1874 data sets with known structures. For each data set the true useful anomalous correlation CC_ano_ was calculated from the observed anomalous differences and the known anomalous substructure. Additionally, the square of the estimated anomalous correlation (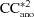), the skew of the anomalous difference Patterson function (*s*) and the estimate of the normalized error in the anomalous differences (*e*) were calculated. For data sets where unmerged data were not available, the expected useful anomalous correlation was not calculated. The Bayesian prior (the information available about the useful anomalous correlation CC_ano_ before making any measurements) was simply the distribution of observations of the useful anomalous correlation in the training set. Given a set of observations (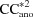, *s* and *e*), the useful anomalous correlation CC_ano_ was estimated by weighting each possible value of CC_ano_ by the prior probability for that value of CC_ano_ multiplied by the probability that the observations 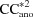, *s* and *e* would have been made if that value of CC_ano_ were correct (Hamilton, 1964 ▸; Terwilliger *et al.*, 2009 ▸). The estimator was trained on all the complete data sets used in this work and separately for data truncated to high-resolution limits from 1.5 to 6 Å in increments of 0.5 Å.

#### 2.3.2. Bayesian estimation of atomic displacement factors for atoms in the substructure   

We developed a similar Bayesian estimator for the atomic displacement factor for atoms in the anomalous substructure based on the Wilson *B* value (Wilson, 1942 ▸) for the entire structure. Using 138 MAD and SAD data sets from the PDB (listed in §[Sec sec2.4]2.4), the Wilson *B* value for the data sets was estimated using the *PHENIX* tool *phenix.xtriage* (Zwart *et al.*, 2005 ▸), and the mean atomic displacement factor for atoms in the anomalous substructure was calculated from the deposited entry in the PDB. A Bayesian estimator was trained as above with a list of Wilson *B* values and corresponding mean atomic displacement factors for the anomalous substructure.

#### 2.3.3. Estimation of the probability of obtaining a correct substructure based on the anomalous signal   

We used a simple approach to estimate the likelihood of obtaining a correct substructure based on values of the anomalous signal. The complete and truncated SAD data sets described above were grouped into bins based on their anomalous signal estimated with the Bayesian estimator just described. The *HySS* likelihood-based substructure-determination method (Bunkóczi *et al.*, 2014 ▸) was applied to each of the data sets and the fraction of sites in the substructure that were obtained (within 3 Å of a correct site) was noted. A substructure was designated as ‘solved’ if 50% or more of the sites in the substructure were obtained. The estimator of the probability of substructure solution for a particular value of anomalous signal (grouped into bins) constructed with these data was then simply the fraction of substructures with this anomalous signal that were solved.

#### 2.3.4. Estimation of the accuracy of phasing based on the useful anomalous correlation   

The same set of SAD data sets and the same approach as in the previous section were used to estimate the quality of initial phases and to compare them with the useful anomalous correlation. For each data set, phase calculations were carried out with *Phaser* (McCoy *et al.*, 2004 ▸) using the known anomalous substructure and the measured anomalous differences for the data set. The resulting map correlation to a model-phased map was then determined and compared with the useful anomalous correlation (*cf.* Fig. 4 in Terwilliger *et al.*, 2016 ▸). The estimator for the expected accuracy of phasing given a value of the useful anomalous correlation was then simply the mean correlation to the model-phased maps for those data sets with similar values of useful anomalous differences (calculated in bins of the useful anomalous difference).

#### 2.3.5. Estimation of anomalous signal before and after collecting the data   

We used (1) to estimate the expected value of the anomalous signal *S*_ano_^obs^ in a crystal structure. This expression requires values of the useful anomalous correlation, the number of reflections and the atomic displacement factors and number of sites in the anomalous substructure.

Even before measuring diffraction intensities, some estimates of these values can be made based on the composition of the crystal. After measuring the data, improved estimates can be made. The useful anomalous correlation CC_ano_ can be estimated after anomalous data are available as described in the previous section. In advance of measuring the data the useful anomalous correlation is not known, but it is related (Fig. 2, below) to the normalized errors in the anomalous differences *e* (11). It is not necessary to know *e* very precisely, as the value of the anomalous signal approaches a maximum value asymptotically as the normalized error decreases (12). In our approach the user can specify the errors in measurement in terms of the anticipated mean value of the ratio of intensity to uncertainty in measurement of intensity 〈*I*/σ(*I*)〉. The empirical relationship of *e* ≃ 0.88/[〈*I*/σ(*I*)〉] (derived from a simple plot of these two variables for 82 of the data sets examined in this work and with a squared Pearson correlation coefficient *r* ^2^ of 0.82 for this relationship) is then used to calculate *e* and to estimate the useful anomalous correlation using the Bayesian estimator described in §[Sec sec2.3.1]2.3.1.

The number of reflections *N*_refl_ is of course known after the data have been measured. In advance of this, it can be estimated from the resolution to which diffraction data are to be measured, the composition of the macromolecule and the solvent content in the crystal. As the high-resolution limit for data collection is generally not known before measuring the data, several values can be examined. The solvent content for many macromolecular crystals is near 50%, although occasionally it may be much higher or lower (Matthews, 1968 ▸; Kantardjieff & Rupp, 2003 ▸; Weichenberger & Rupp, 2014 ▸). We used the *PHENIX* tool *phenix.ncs_and_number_of_ha* to estimate the composition of the asymmetric unit of the crystal and the solvent content. From the resulting estimate of the volume *V*_au_ of the asymmetric unit in the crystal and the resolution *d*_min_ to which data are to be collected, the number of unique (non-anomalous) reflections can be estimated as (following Ladd, 1998 ▸ and noting the caveat that this calculation includes centric reflections that will not contribute to anomalous scattering)
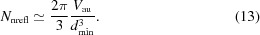
The number of sites in the anomalous substructure can often be estimated from the sequence of the macromolecule, for example in cases in which the anomalous substructure is part of the macromolecule, such as in selenomethionine or sulfur SAD experiments (Dauter, 2006 ▸). In other cases such as soaks with noncovalent heavy-atom derivatives it may be necessary to guess the number of sites. We use the *PHENIX* tool *phenix.ncs_and_number_of_ha* to estimate the number of sites.

The mean atomic displacement factor (needed to calculate the second moment of the scattering factors *f_B_* with equation 2) for the atoms in the substructure is generally not known in advance of measuring anomalous data, and in this case a Bayesian estimate of its value is made based on the anticipated resolution of data collection and the values of atomic displacement factors for the anomalous substructure and resolution for the 248 data sets used in §[Sec sec2.3.2]2.3.2 (these data are available in the *PHENIX* software in the file $PHENIX/modules/cctbx/mmtbx/scaling/ha_b_from_wilson.dat). Once the data have been measured it could in principle be estimated from the fall-off with resolution of anomalous differences, corrected for errors in measurement. We have instead calculated the atomic displacement factors for the anomalous substructure from the Wilson *B* value of the data as a whole using a simple Bayesian estimator as described in §[Sec sec2.3.2]2.3.2.

### 2.4. Test data from the PDB   

We downloaded data sets from the PDB to serve as test cases for our analyses. The data consisted of 218 MAD and SAD data sets from 113 different PDB entries with diffraction data extending to resolutions from 1.2 to 4.5 Å and with anomalously scattering atoms including selenium, sulfur, cobalt, mercury, zinc, nickel, iron, calcium, barium and iridium. The MAD PDB entries were split into individual data sets for each wavelength of X-ray data used to measure diffraction data. The *PHENIX* tool *phenix.sad_data_from_pdb* was used to extract the individual data sets from PDB entries. The PDB entries used were 1vjn, 1vjr, 1vjz, 1vk4, 1vkm (Levin *et al.*, 2005 ▸), 1vlm, 1vqr (Xu *et al.*, 2006 ▸), 1xri (Aceti *et al.*, 2008 ▸), 1y7e, 1z82, 1zy9, 1zyb, 2a2o, 2a3n, 2a6b, 2aj2, 2aml, 2avn, 2b8m, 2etd, 2etj, 2ets (Kozbial *et al.*, 2008 ▸), 2etv, 2evr (Xu, Sudek *et al.*, 2009 ▸), 2f4p, 2fea (Xu *et al.*, 2007 ▸), 2ffj, 2fg0 (Xu, Sudek *et al.*, 2009 ▸), 2fg9, 2fna (Xu, Rife *et al.*, 2009 ▸), 2fqp, 2fur, 2fzt, 2g0t, 2g42, 2gc9, 2nlv (Hwang *et al.*, 2014 ▸), 2nuj, 2nwv, 2o08, 2o1q, 2o2x, 2o2z, 2o3l, 2o62, 2o7t, 2o8q, 2obp, 2oc5, 2od5, 2od6, 2oh3, 2okc, 2okf (Hwang *et al.*, 2014 ▸), 2ooj, 2opk, 2osd, 2otm, 2ozg, 2ozj, 2p10, 2p4o, 2p7h, 2p7i, 2p97, 2pg3, 2pg4, 2pgc, 2pim, 2pn1, 2pnk, 2ppv, 2pr1, 2pr7, 2prv, 2pv4, 2pw4, 2wcd (Mueller *et al.*, 2009 ▸), 2xdd (Fineran *et al.*, 2009 ▸), 2zxh (Osawa *et al.*, 2009 ▸), 3caz, 3din (Zimmer *et al.*, 2008 ▸), 3dto, 3fx0 (Lo *et al.*, 2009 ▸), 3guw, 3gw7, 3hxk, 3hxp, 3lml, 3mv3 (Hsia & Hoelz, 2010 ▸), 3ov0 (Pokkuluri *et al.*, 2011 ▸), 3pg5, 3zgx (Bürmann *et al.*, 2013 ▸), 3zxu (Schmitzberger & Harrison, 2012 ▸), 4acb (Leibundgut *et al.*, 2004 ▸), 4asn (Aylett & Lowe, 2012 ▸), 4bql (Lindås *et al.*, 2014 ▸), 4cb0 (Mechaly *et al.*, 2014 ▸), 4cbv (Boudes *et al.*, 2014 ▸), 4fsx (Du *et al.*, 2012 ▸), 4g9i (Tominaga *et al.*, 2012 ▸), 4gkw (Qiao *et al.*, 2012 ▸), 4h6y (He *et al.*, 2013 ▸), 4hkr (Hou *et al.*, 2012 ▸), 4hnd (Zhou *et al.*, 2014 ▸), 4ifq (Sampathkumar *et al.*, 2013 ▸), 4lck (Zhang & Ferré-D’Amaré, 2013 ▸), 4nsc (Wang *et al.*, 2014 ▸), 4nt5 (Zhou & Springer, 2014 ▸), 4px7 (Fan *et al.*, 2014 ▸), 4q8j (Schäfer *et al.*, 2014 ▸), 4qka (Gao & Serganov, 2014 ▸) and 4tq5 (Huang *et al.*, 2014 ▸).

For the estimation of the atomic displacement factors for the atoms in the anomalous substructure in §[Sec sec2.3.2]2.3.2, an additional 25 PDB entries were used. These were 1hf8 (Ford *et al.*, 2001 ▸), 2ahy (Shi *et al.*, 2006 ▸), 2b78, 2fdn (Dauter *et al.*, 1997 ▸), 2hba (Cho *et al.*, 2014 ▸), 2o0h (Sun *et al.*, 2007 ▸), 2prx, 2wxw (Zhou *et al.*, 2010 ▸), 3fki (Meyer *et al.*, 2009 ▸), 3i5d (Kawate *et al.*, 2009 ▸), 3iko (Nagy *et al.*, 2009 ▸), 3k9g (Abendroth *et al.*, 2011 ▸), 3km3 (Abendroth *et al.*, 2011 ▸), 3m6a (Duman & Löwe, 2010 ▸), 3p96 (Abendroth *et al.*, 2011 ▸), 3qqc (Martinez-Rucobo *et al.*, 2011 ▸), 4a2n (Yang *et al.*, 2011 ▸), 4ai6 (Schmidt *et al.*, 2012 ▸), 4aj5 (Jeyaprakash *et al.*, 2012 ▸), 4aki (Schmidt *et al.*, 2012 ▸), 4b09 (Choudhury & Beis, 2013 ▸), 4biu (Mechaly *et al.*, 2014 ▸), 4cv5 (Mathys *et al.*, 2014 ▸), 4m2s (Li *et al.*, 2014 ▸) and 4nha (Tian *et al.*, 2014 ▸).

## 3. Results and discussion   

### 3.1. Scaling and merging of anomalous data from one or more data sets while explicitly modeling inter-data-set variation   

Our procedure for scaling and merging SAD data has three key elements. Firstly, our procedure uses local scaling (Matthews & Czerwinski, 1975 ▸; Terwilliger & Berendzen, 1999 ▸) to minimize systematic differences between members of Bijvoet pairs. Secondly, our procedure explicitly models the differences between the data coming from different data sets as a data-set-specific variance and weights the data from different data sets accordingly. Thirdly, this data-set variance is calculated based on anomalous differences, not the Bijvoet-averaged amplitudes, so that the weighting of data sets optimizes the anomalous differences, not the overall amplitudes.

Modeling the differences in data coming from different data sets is essentially a way to replace the standard procedure of outlier rejection with a method for down-weighting data coming from data sets that are systematically different from an average data set. For each data set (within a bin of resolution), the anomalous differences measured for that data set are compared with anomalous differences obtained by averaging all data sets. This yields an estimate of the total variance between that data set and the mean. Part of this total variance can normally be accounted for by the experimental uncertainties for the data set in question and from the estimated errors in the averaged values. In our approach, the remaining (unexplained) variance is considered to be inter-data-set variation. This inter-data-set variance represents how different this data set is from the average of all data sets. If the data sets correspond to different crystals, the inter-data-set variance is an estimate of how different the data obtained from this crystal would be from data averaged over all crystals if all the data were measured without error. Including this inter-data-set variance in the weighting of the data during merging has the effect of automatically reducing the contribution of data sets that are very different from the others, but still including an appropriate amount of information from these data sets.

In a SAD experiment, it is normally the accuracy of the anomalous differences, rather than the accuracy of the amplitudes obtained after averaging Bijvoet pairs of reflections to remove the anomalous contribution, that is most critical for structure determination. In cases where there is substantial inter-data-set variation, the variation between data sets in Bijvoet-averaged amplitudes is not necessarily the same as the variation in anomalous differences. For example, a set of data sets might be collected at slightly different X-ray wavelengths but otherwise be essentially identical. In this case the inter-data-set variation for Bijvoet-averaged pairs might be very small, but the variation for anomalous differences might be very large. To obtain the most accurate estimate of anomalous differences at one X-ray wavelength, then, it would be important to down-weight data sets collected at other X-ray wavelengths. This can be accomplished by using the anomalous differences themselves in the estimation of the inter-data-set variation.

### 3.2. Utility of modeling inter-data-set variation when merging anomalous data   

**Figure 1:**
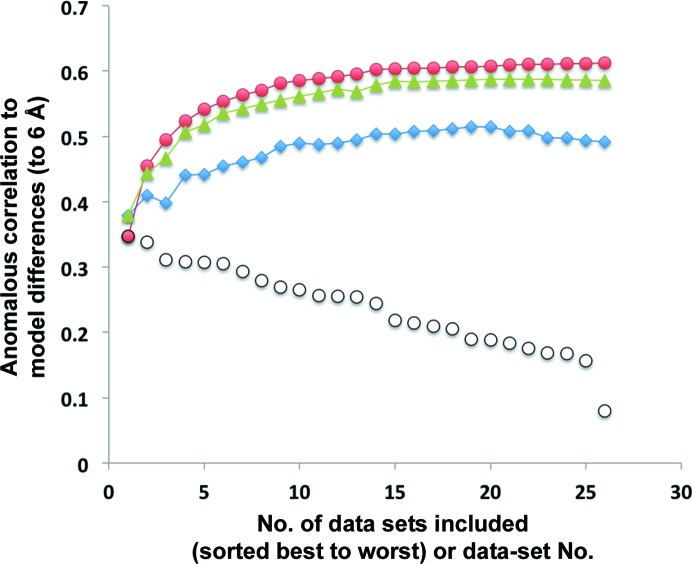
Useful anomalous correlation for NS1 merged data sets. Flavivirus NS1 data sets were individually scaled and merged. Anomalous differences for each individually merged data set were compared with model anomalous differences based on the deposited structure (PDB entry 4tpl) to yield values of the useful anomalous correlation for individual data sets (black open circles). The individually merged data sets were then combined using *phenix.scale_and_merge* in three ways: modeling inter-data-set variances based on anomalous differences (red closed circles), modeling inter-data-set variances based on averaged Bijvoet pairs (green closed triangles) or without modeling inter-data-set variances (blue closed diamonds).

Fig. 1 ▸ shows the utility of explicitly modeling inter-data-set variation in merging of data from multiple crystals. The data are taken from an analysis carried out by Akey *et al.* (2014 ▸) on data from 28 crystals of the flavivirus NS1 protein. As the structure of the NS1 protein is available (PDB entry 4tpl), we were able to calculate expected (ideal) anomalous differences from the known structure based on anomalous contributions from the S atoms in the structure. These ideal anomalous differences were compared with the anomalous differences from individual data sets and from merging various groups of data sets, and the correlation of anomalous differences (the useful anomalous correlation, CC_ano_) was used as a measure of the quality of the experimentally determined and merged anomalous differences. Using the useful anomalous correlation as a metric, we ordered 26 of the 28 data sets from high (0.35) to low (0.08) values of useful anomalous correlation using data to a resolution of 6 Å (shown in Fig. 1 ▸). We then created merged data sets containing from one to all 26 of these individual data sets and scaling in one of three ways. The first approach included modeling inter-data-set variation and optimization of anomalous differences. The second included modeling inter-data-set variation, but calculating the inter-data-set variation using Bijvoet-averaged pairs instead of with the anomalous differences, and the third used neither of these methods. Fig. 1 ▸ shows that if no modeling of inter-data-set variation is carried out at all, anomalous differences for the first data set have a correlation with the model anomalous differences of 0.38. Adding additional data sets with decreasing useful anomalous correlation initially increases the useful anomalous correlation of the merged data (up to a maximum of 0.52), but as very poorly correlated data sets are added the anomalous differences for the merged data set becomes less correlated with the model anomalous differences, so that when all 26 data sets are merged the correlation is 0.49. We then examined the effect of including inter-data-set modeling of variance based on the anomalous differences. In this case the correlation of anomalous differences for the merged data continually increases with additional data sets, even when the added data sets have very low correlation with the model anomalous differences. When all data sets are included, the correlation of anomalous differences with model differences is 0.61. Finally, we examined the importance of modeling the inter-data-set variation using anomalous differences. When the inter-data-set variation was based instead on the inter-data-set variation of Bijvoet-averaged structure-factor amplitudes [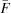 = ½(*F* ^+^ + *F* ^−^)], the correlation of merged anomalous differences with model differences were consistently slightly lower than when modeling the inter-data-set variation on the anomalous differences (Δ_ano_ = *F* ^+^ − *F* ^−^), but otherwise the results were quite similar. When all data sets were included in this case, the correlation with model anomalous differences was 0.59.

### 3.3. Estimating the useful anomalous correlation in a SAD data set after measurement of anomalous data   

It has been shown in Terwilliger *et al.* (2016 ▸) that the anomalous signal and the useful anomalous correlation are useful indicators of the utility of the anomalous differences in a SAD data set. These metrics of data quality can only be calculated directly after a structure has been solved, so methods for estimating them from measured data would be valuable. In this section, we will focus on estimating the useful anomalous correlation using three measurable quantities available once the data have been collected: the half-data-set anomalous correlation, the skew of the anomalous difference Patterson and the normalized error in measurement of the anomalous differences.

#### 3.3.1. Relationship between useful anomalous correlation and half-data-set anomalous correlation   

A measure of the utility of anomalous differences is the half-data-set anomalous correlation (CC_ano_^half^; Evans, 2006 ▸). A high value of the half-data-set anomalous correlation indicates that the anomalous differences can be reproducibly measured. Taking into consideration the number of observations of each reflection in each half data set, it is possible to use the half-data-set anomalous correlation CC_ano_^half^ to estimate how similar the merged anomalous differences for a data set are to the merged differences that would be obtained if infinitely many observations of them were made (Karplus & Diederichs, 2012 ▸; equation 7). This estimated anomalous correlation 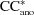 is essentially a measure of how close the measured anomalous differences are to the true anomalous differences for this crystal. It is not, however, necessarily a measure of how close the anomalous differences are to those that would be obtained from an idealized crystal in which the only scatterers are those in the anomalous substructure. Accurately measured anomalous differences can also reflect the anomalous scattering from minor sites for the principal anomalous scatterers in the structure as well as scattering from all of the weak anomalous scatterers in the structure.

**Figure 2:**
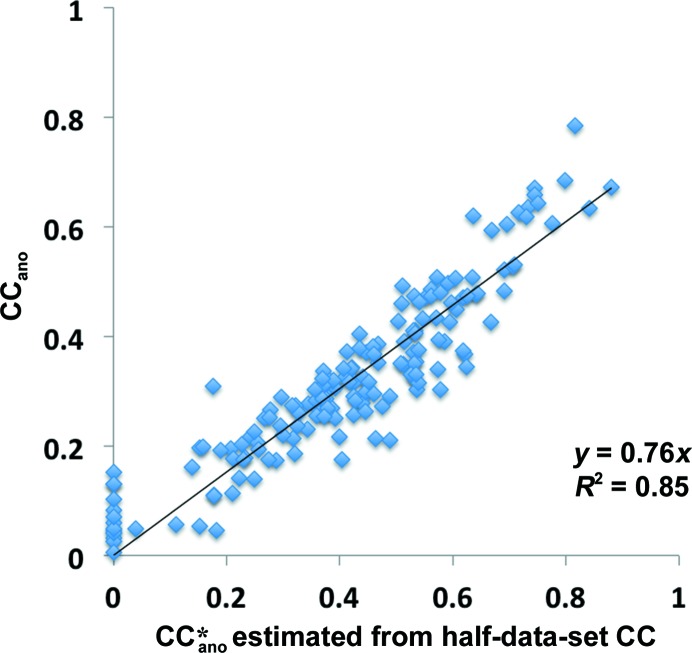
Useful anomalous correlation as function of estimated anomalous correlation. The useful anomalous correlation is calculated from the correlation of model-based and measured anomalous differences. The estimated anomalous correlation is calculated from the half-data-set anomalous correlation using (7).

Fig. 2 ▸ shows the relationship between the estimated anomalous correlation 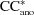 and the useful anomalous correlation CC_ano_^obs^ for the subset of SAD data sets listed in §[Sec sec2.4]2.4 where unmerged data are available. The estimated anomalous correlation is very closely related to the useful anomalous correlation (squared Pearson correlation coefficient *r* ^2^ of 0.85). It can be seen, however, that the useful anomalous correlation is consistently less than the estimated anomalous correlation (the slope of the least-squares fit of CC_ano_^obs^ to 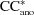 in Fig. 2 ▸ is about 0.75). This suggests that for this group of SAD data sets (and presumably others as well) there are systematic differences between the measured anomalous differences and those calculated from the final model for the structure and sub­structure. These differences could come from systematic errors in measurement, but another likely source of such systematic differences would be that a substantial fraction of the anomalous scattering is owing to anomalous scattering not from the substructure itself. Extrapolation of the relationship shown in Fig. 2 ▸ suggests that even if anomalous differences were measured perfectly in these data sets, the useful anomalous correlation would be only about 0.75.

We note that obtaining the relationship shown in Fig. 2 ▸ requires careful scaling and grouping of the anomalous data. Single-wavelength anomalous data (SAD data) are often collected from multiple crystals or from multiple orientations of a single crystal. The various crystals may differ slightly and even a single crystal may change during data collection. Consequently, the scaling and merging of the data for a SAD experiment is an important step in solving the structure. In calculating the half-data-set anomalous correlation, all of the data taken from the PDB were first split into half data sets and rescaled using the *phenix.scale_and_merge* algorithm described in §[Sec sec2.1]2.1. When half-data-set anomalous correlations were calculated using data from the PDB without rescaling and choosing half data sets randomly, the squared Pearson correlation coefficient *r* ^2^ for the relationship between CC_ano_^obs^ and 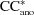 was only 0.37 (rather than 0.85 as in Fig. 2 ▸).

#### 3.3.2. Relationship between useful anomalous correlation and the skew of the anomalous difference Patterson   

**Figure 3:**
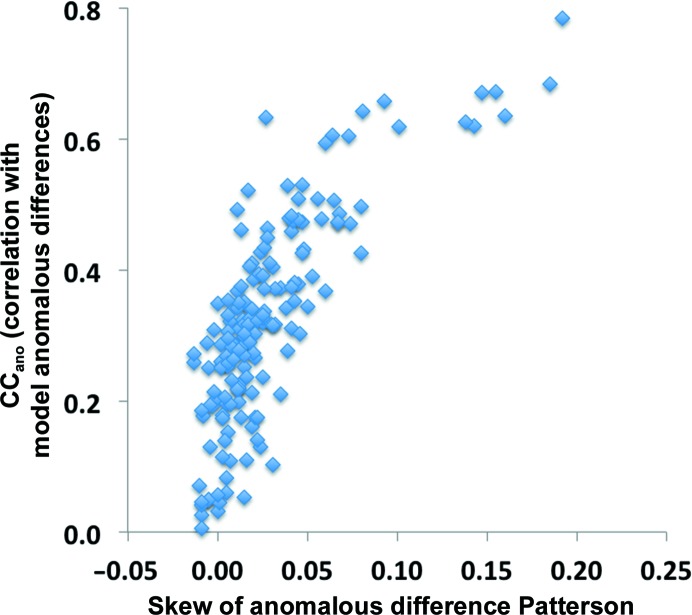
The skew of the anomalous difference Patterson function is calculated as described in §[Sec sec2.2.2]2.2.2 (9).

Although the estimated anomalous correlation (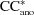) as estimated from the half-data-set anomalous correlation (CC_ano_^half^) is a very good indicator of the useful anomalous correlation (CC_ano_^obs^), in cases where multiple measurements of anomalous differences have not been made or are not available it is helpful to have other metrics that are related to the useful anomalous correlation. One of these that we have used is the skew of the anomalous difference Patterson. The skew of the values in a map is generally positive if there are many positive peaks in the map and few negative ones, as should be the case for a useful anomalous difference Patterson function. We calculate the skew of a map after truncating very high and very low values (see §[Sec sec2.2.2]2.2.2). Fig. 3 ▸ shows the relationship between the skew (*s*) of anomalous difference Patterson functions calculated using the SAD data sets listed in §[Sec sec2.2]2.4 and the useful anomalous correlation (CC_ano_^obs^). It can be seen that in general data sets that have a high useful anomalous correlation have a high skew as well.

#### 3.3.3. Relationship between useful anomalous correlation and estimates of the normalized error in anomalous differences   

**Figure 4:**
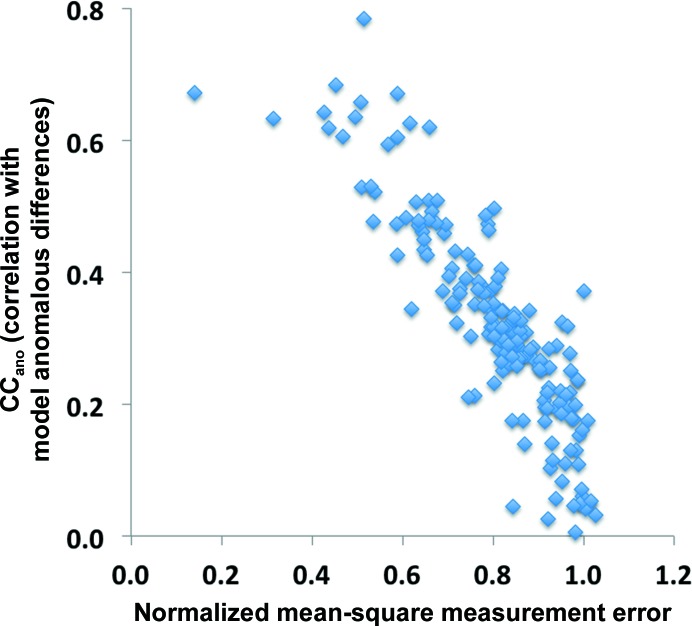
Useful anomalous correlation as a function of estimated error in anomalous differences. The useful anomalous correlation is as in Fig. 2 ▸. The normalized error in the anomalous differences is calculated as described in §[Sec sec2.2.3]2.2.3 (11).

A third indicator of the useful anomalous correlation that we have used here is an estimate of the normalized error in measured anomalous differences. It is difficult to obtain accurate estimates of uncertainties in anomalous differences (or in intensity measurements; Dauter, 2006 ▸; Evans, 2006 ▸). Here, we have made the assumption that error estimates for a particular SAD data set might be proportionally smaller or larger than the true errors, and we have adopted a procedure designed to find this scale factor. Our procedure (see §[Sec sec2.2.3]2.2.3) is simply to assume that at the highest resolution where data were collected there is essentially no anomalous signal, so that the anomalous differences are entirely noise. Using this assumption, it is simple to rescale all of the uncertainties in a data set and to calculate the ratio of the r.m.s. uncertainty in measurement to the r.m.s. anomalous difference, which we term the normalized error estimate (11). Fig. 4 ▸ shows that these normalized error estimates are indeed inversely related to the useful anomalous correlation, where the normalized error estimates are small for cases where the useful anomalous correlation is high, and is about one for cases where the anomalous signal is near zero.

#### 3.3.4. Bayesian estimation of the useful anomalous correlation after obtaining anomalous data   

**Figure 5:**
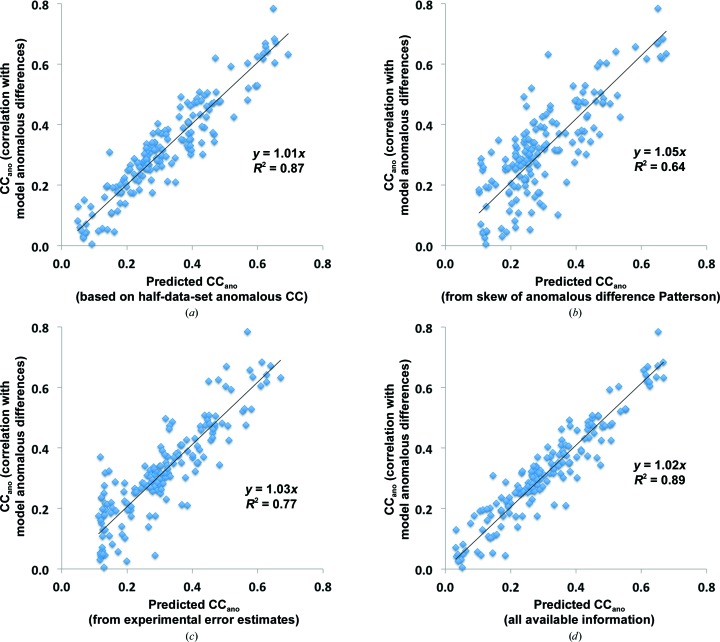
Cross-validation of Bayesian estimates of useful anomalous correlation. The Bayesian estimator described in §[Sec sec2.3.1]2.3.1 was used to generate estimates of the useful anomalous correlation based on (*a*) the half-data-set anomalous correlation, (*b*) the skew of the anomalous difference Patterson, (*c*) the normalized error in the anomalous differences and (*d*) all three. The data used are from the 166 SAD data sets in Fig. 2 ▸ where unmerged data were available so that all three measures were available. To carry out the cross-validation the data set to be analysed was left out of the training set for the estimator. The *x* coordinate for each point is the value of the useful anomalous correlation from the Bayesian estimator for one SAD data set and the *y* coordinate is the actual value of the useful anomalous correlation for that data set.

We created a simple Bayesian estimator (§[Sec sec2.3.1]2.3.1) that uses information from three sources, the half-data-set anomalous correlation, the skew of the anomalous difference Patterson function and the normalized error in the anomalous differences, to predict the useful anomalous correlation. To evaluate the utility of this estimator, we carried out a cross-validation analysis in which one data set was excluded at a time in the training of the estimator and this version of the estimator was then used to predict the value of the useful anomalous correlation for this data set. We carried out this analysis for estimators created with each source of information separately and for all three sources together. Fig. 5 ▸ illustrates this cross-validation analysis. The squared Pearson correlation coefficient (*r* ^2^) between the predicted and observed useful anomalous correlation ranged from 0.64 based on the skew of the anomalous difference Patterson function (Fig. 5 ▸ *b*) to 0.77 based on estimates of the normalized error in measurement (Fig. 5 ▸ *c*) to 0.87 using the half-data-set anomalous correlation (Fig. 5 ▸ *a*) and 0.89 using all of these measures together (Fig. 5 ▸ *d*). This analysis indicates that it is possible to obtain quite accurate estimates of the quality of anomalous differences (their useful anomalous correlation) based on simple measures that can be calculated from the measured data. If unmerged data are available so that the half-data-set anomalous correlation can be calculated, estimates of the useful anomalous correlation can be particularly accurate (Figs. 5 ▸ *a* and 5 ▸ *d*).

### 3.4. Planning a SAD experiment and evaluating a SAD data set   

As shown in Terwilliger *et al.* (2016 ▸), the anomalous signal in a SAD data set is a very good predictor of whether the anomalous substructure can be determined using likelihood-based methods. The anomalous signal in turn is a simple function of the useful anomalous correlation, the number of unique reflections, the number of sites in the anomalous substructure and the atomic displacement factors for the anomalous substructure (1). If estimates can be made of these factors, then it would be possible to estimate the anomalous signal in a SAD data set and subsequently the probability of finding the anomalous substructure. We have developed simple tools for carrying out this estimation of the anomalous signal and the probability of determining the anomalous substructure both before and after measuring the diffraction data.

#### 3.4.1. Planning a SAD experiment   

Estimates of the anomalous signal for a SAD experiment before measuring the diffraction data would be of considerable use in planning an experiment. If the number of atoms in the anomalous sub­structure were known and the atomic displacement factors could be guessed, it would be possible to identify how many reflections would have to be measured to find the substructure if the data were measured perfectly (1). Further, our Bayesian approach for estimation of the useful anomalous correlation CC_ano_^obs^ from the normalized error in anomalous differences *e* (§[Sec sec2.2.3]2.2.3) could be used to identify how accurately the anomalous differences would need to be measured to find the substructure with real data.

We have created a *PHENIX* tool, *phenix.plan_sad_experiment*, that estimates the anomalous signal in a SAD experiment in advance of collecting the anomalous data (see §[Sec sec2.3.5]2.3.5). The tool uses the composition of the macromolecule in the crystal, any available information about the type and number of anomalous scatterers and the wavelength of planned data collection. The approach then uses (1) to estimate, for various high-resolution limits in data collection, how accurately the data would have to be measured (what value of mean intensity divided by uncertainty in intensity) in order to achieve an anomalous signal of about 15 or greater (if such a signal can be achieved at all). The tool uses the Bayesian estimator introduced in §[Sec sec2.3.3]2.3.3 to calculate the probability of determining the anomalous substructure with likelihood-based methods using data to these various resolution limits and measured with this accuracy. The tool also estimates the accuracy of phasing that could be expected if the substructure is solved (§[Sec sec2.3.4]2.3.4).

**Figure 6:**
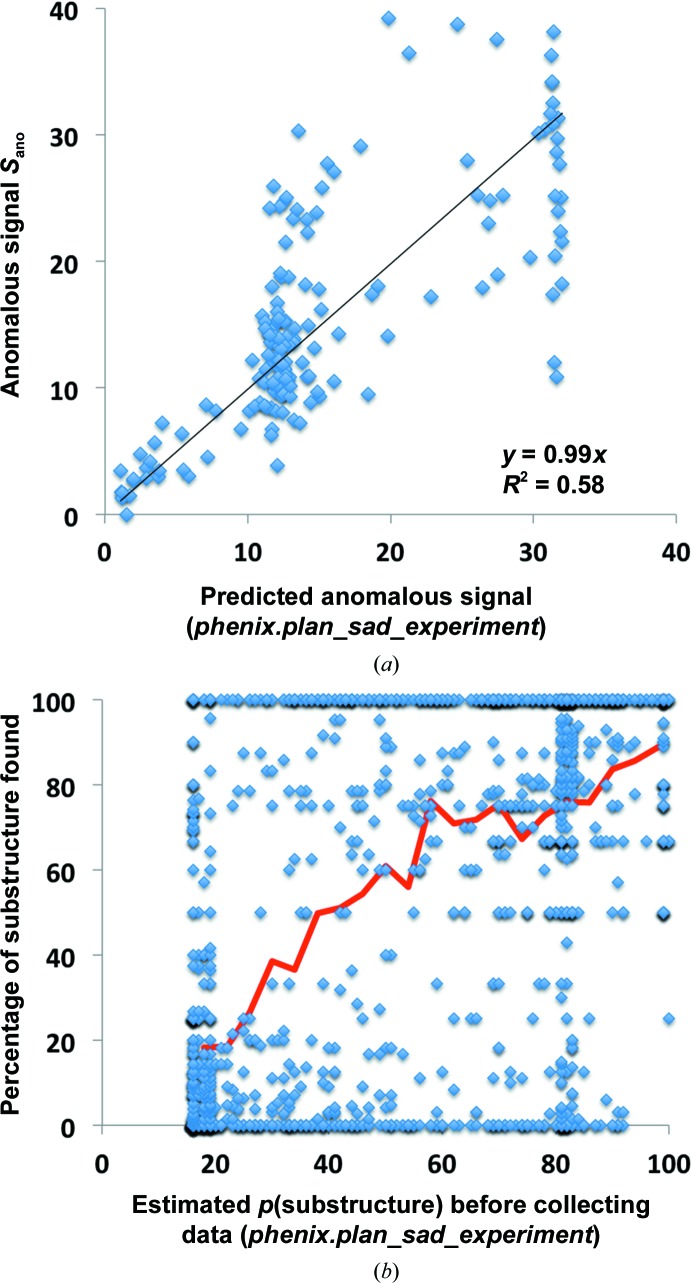
(*a*) Anomalous signal predicted using information available before the measurement of diffraction data for SAD data sets. The 218 SAD data sets are those shown in Fig. 3 ▸. The anomalous signal was predicted from the composition of the crystal, the wavelength of data collection and the resolution of data collection as described in the text. The actual anomalous signal is the normalized value of the anomalous difference Fourier map at coordinates of atoms in the anomalous substructure. (*b*) The fraction of the substructure correctly determined by likelihood-based substructure search as a function of the probability of structure solution estimated using information available before measurement of diffraction data. Each point is the fraction of correct sites for one SAD data set. The line is the smoothed average.

Fig. 6 ▸ illustrates the accuracy of the overall predictions that could have been obtained by using the *phenix.plan_sad_experiment* tool on the 218 SAD data sets listed in §[Sec sec2.4]2.4 before measuring the diffraction data. The information provided to the tool consisted of the sequence of the macromolecule, the identity of the anomalously scattering atom, the wavelength of data collection and the high-resolution limit of data collection. The high-resolution limit was provided so that the tool would estimate the anomalous signal at a resolution matching the actual data, but we note that this did provide some information to the tool that might not be available in a real situation. The high-resolution limit was used to estimate the atomic displacement factors for the anomalously scattering atoms. The number of sites in the anomalous substructure was not provided to the tool but was instead guessed from the sequence and the type of anomalous scatterer using the *phenix.ncs_and_number_of_ha* method in *PHENIX*. The *phenix.plan_sad_experiment* tool was then used to predict the anomalous signal and the probability of successful substructure determination. Fig. 6 ▸(*a*) shows the predicted and actual anomalous signal for each SAD data set. For most of the data sets the actual anomalous signal was within about a factor of two of the predicted signal, and the squared Pearson correlation coefficient 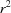 for this prediction is 0.58. Fig. 6 ▸(*b*) shows the fraction of substructure atoms correctly identified for each SAD data set as a function of the estimated probability of successful substructure determination. The (smoothed) mean fraction of sites found is plotted as a solid line in Fig. 6 ▸(*b*), and it can be seen that the estimated probability of success is fairly close to the actual success rate. The reason the lowest values of predicted success are not zero, but rather are about 20%, is that there are some cases where the anomalous signal estimated before collecting any data was very low but there was significant anomalous signal and the substructure could still be determined.

#### 3.4.2. Analyzing a SAD data set and estimating the probability of substructure solution and expected accuracy in phasing   

Once SAD data have been collected, improved estimates of the useful anomalous correlation and anomalous signal can be obtained because several of the factors in (1) can be estimated with increased accuracy. The Bayesian estimator described in §[Sec sec2.3.1]2.3.1 can be applied to the half-data-set anomalous correlation, the skew in the anomalous difference Patterson function and the normalized error estimate for the anomalous differences to obtain a realistic estimate of the correlation CC_ano_^obs^ between measured anomalous differences and those expected for an ideal structure where anomalous differences come only from atoms in the substructure. Fig. 5 ▸(*d*) illustrates a cross-validation analysis of predicted and actual useful anomalous correlation calculated in this way for those data sets where all three sources of information are available (*i.e.* those for which unmerged data are available so that the half-data-set anomalous correlation can be obtained). The squared Pearson correlation coefficient *r* ^2^ for this relationship was 0.89.

**Figure 7:**
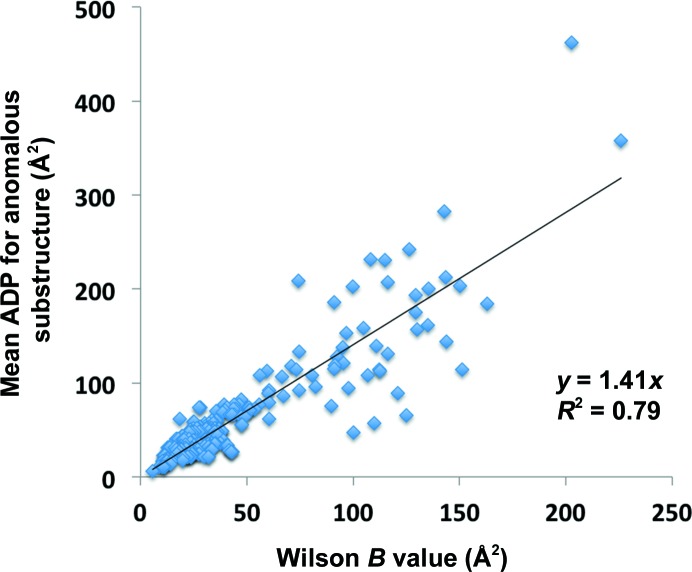
Mean atomic displacement factor for atoms in the anomalous sub­structure as a function of the Wilson *B* value (see text for details).

Once the data have been collected, the number of unique reflections *N*_refl_ is of course known. Additionally, some information becomes available about the atomic displacement factors for the anomalous substructure. The mean atomic displacement factors could in principle be estimated from the anomalous differences and the errors in measurement. In this work, we simply estimate them from the Wilson *B* value (overall atomic displacement factor) for the measured data (Wilson, 1942 ▸) as described in §[Sec sec2.3.2]2.3.2. The relationship between the overall Wilson *B* value and mean atomic displacement factor for the anomalous substructure is shown in Fig. 7 ▸. It can be seen that the mean atomic displacement factor for the anomalous substructure is larger than the Wilson *B* value by about 40% on average, although there is considerable variation (the squared Pearson correlation coefficient *r* ^2^ for the linear fit shown is 0.79).

**Figure 8:**
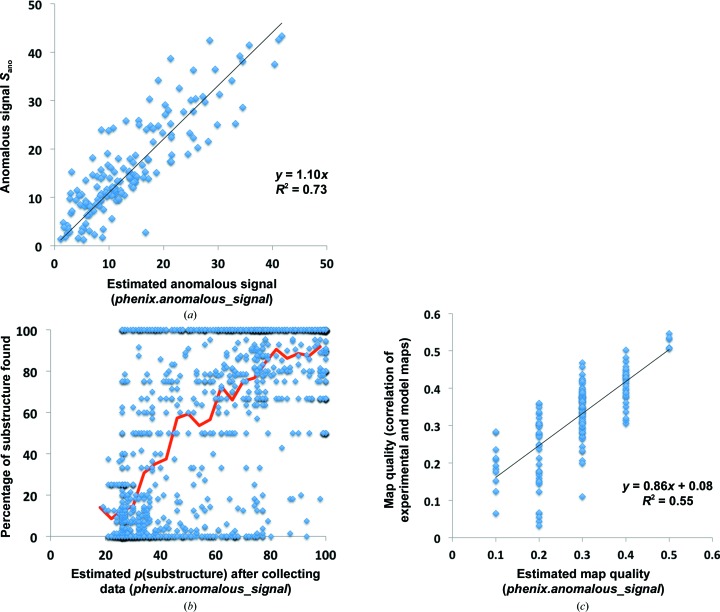
(*a*) Anomalous signal estimated after measurement of X-ray data. The anomalous signal for each SAD data set was estimated as described in the text using the values of useful anomalous correlation in Fig. 5 ▸(*d*) and (1). (*b*) Fraction of the substructure correctly determined by likelihood-based substructure search as a function of the estimated probability of structure solution, as in Fig. 6 ▸(*b*), except that the predictions are made after obtaining the X-ray data. (*c*) Estimation of the experimental map quality obtainable once the substructure has been determined based on estimates of useful anomalous correlation. Map quality is defined here as the correlation of values at grid points in the experimental map with the corresponding values in a 2*mF*_o_ − *DF*_c_ σ_A_-weighted map (Read, 1986 ▸) based on a final refined model.

Fig. 8 ▸(*a*) illustrates calculations of the anomalous signal obtained with (1) for all data sets using estimates of the useful anomalous correlation, the number of reflections and atomic displacement factors for the substructure available after collecting the data. The squared Pearson correlation coefficient *r* ^2^ for the linear fit shown is 0.73. This prediction is less accurate than that shown in Fig. 5 ▸(*d*) for the useful anomalous correlation. This is partly because the anomalous signal depends on the number of sites and the atomic displacement factors for the sites in the substructure, each of which are known only approximately. The lower accuracy of the prediction of the anomalous signal in Fig. 8 ▸(*a*) compared with the useful anomalous correlation shown in Fig. 5 ▸(*d*) is also owing to the fact that all data sets from §[Sec sec2.4]2.4 are included in Fig. 8 ▸(*a*) but data sets without unmerged data are excluded in Fig. 5 ▸(*d*).

Using the anomalous signal estimates available after collecting the data from Fig. 8 ▸(*a*), we estimated the probability of successful substructure determination as described in §[Sec sec2.3.3]2.3.3. Fig. 8 ▸(*b*) illustrates the actual fraction of substructure sites found as a function of this estimated probability. Comparing Fig. 8 ▸(*b*) with Fig. 6 ▸(*b*), it can be seen that the discrimination between data sets that are likely to lead to a successful substructure determination has been improved by using the data in the prediction. The squared Pearson correlation coefficient *r* ^2^ for the relationship between predicted success in substructure determination and the actual fraction of sites found increases from 0.34 to 0.48.

Once data have been collected and the useful anomalous correlation CC_ano_^obs^ has been estimated, it is possible to obtain improved estimates of the quality of experimental phasing that can be obtained if the substructure is determined. Fig. 8 ▸(*c*) compares estimates and actual values of the accuracy of experimental phasing, as measured by the correlation between experimentally phased electron-density maps and the corresponding maps calculated using a refined model.

## 4. Conclusions   

There are two crucial steps in structure determination using SAD phasing. The first is determining the locations of the atoms in the substructure and the other is the estimation of crystallographic phases (Liu *et al.*, 2013 ▸). Empirical studies have shown that the two steps depend on two related but different aspects of the data in a SAD experiment. Success in substructure solution is most closely related to the anomalous signal (Bunkóczi *et al.*, 2014 ▸; Terwilliger *et al.*, 2016 ▸). The anomalous signal is the mean peak height at coordinates in a model-phased anomalous difference Fourier, and as such is a measure of the overall information about each anomalously scattering atom in the structure. Consequently, it is not surprising that the ability to find the locations of these atoms is closely related to the anomalous signal. In contrast, the quality of experimental phases in the SAD method is found to be most closely related to the useful anomalous correlation CC_ano_^obs^ (Terwilliger *et al.*, 2016 ▸). This observation is also not surprising because the quality of experimental phases is dependent on how closely the measured anomalous differences represent the useful anomalous differences corresponding to the atoms in the substructure, and the useful anomalous correlation describes this similarity. This analysis is consistent with the observations of Zwart (2005 ▸) and provides a basis for predicting experimental map quality before and after collecting SAD data. The anomalous signal is related to the useful anomalous correlation in a simple way (1) that depends on the square root of the number of reflections divided by the number of sites in the anomalous substructure and the second moment of the scattering factors of the anomalously scattering atoms. Before the collection of the data, an upper limit on the value of the useful anomalous correlation can be estimated from the anticipated errors in measurement. In this work, we add an additional step and calculate an expected value of the useful anomalous correlation with a Bayesian estimator (§[Sec sec2.3.1]2.3.1) trained on the uncertainties in measurement and useful anomalous correlations found in the SAD data sets examined here. After measurement of X-ray data, much better estimates of the useful anomalous correlation can be obtained. In particular, the estimates of uncertainties in measurement obtained from the experiment, calculations of the skewness of the anomalous difference Patterson function obtained from the anomalous data and calculation of the half-data-set anomalous correlation all provide substantial information about the useful anomalous correlation. As the anomalous signal is related to the anomalous correlation through (1), after collection of the X-ray data both can be estimated much more accurately than before carrying out the experiment. Consequently, both the probability of substructure determination and the expected accuracy of experimental phases can be estimated more accurately after collection of the data (*cf.* Fig. 6 ▸ *versus* Fig. 8 ▸).

The *phenix.scale_and_merge* software tool described here is useful for carrying out local scaling on one or more anomalous data sets and merging the resulting scaled data sets into a single data set with estimates of the intensities of each unique reflection and its Bijvoet mate. This tool is designed to take data-set-specific differences explicitly into account, so that if a few data sets are systematically different from all the others, intensities from those few data sets are included with much lower weights and are effectively excluded from the analysis. The *phenix.plan_sad_experiment* and *phenix.anomalous_signal* tools both carry out estimation of the probability of finding the substructure and of the quality of experimental phases expected in a SAD experiment. The *phenix.plan_sad_experiment* tool only uses information available before carrying out the experiment and the *phenix.anomalous_signal* tool takes advantage of the information obtained from the experiment itself to improve the estimates. Taken together, these tools provide a practical implementation of our theor­etical analysis of the anomalous signal and useful anomalous correlation in data from a SAD experiment and their implications for structure determination.

Finding the substructure of anomalously scattering atoms and calculating an initial electron-density map with a particular phase accuracy does not in itself define whether a structure can be solved. This work could be extended by developing an estimator of the probability of structure solution based on prediction of map quality combined with solvent content and noncrystallographic symmetry present in the crystal. Our Bayesian estimator linking the anomalous signal in a data set to the likelihood of substructure solution could potentially be integrated into data-processing procedures optimizing integration parameters, selecting the most useful sub-data sets from radiation-damaged data and combining multiple data sets to produce optimized data for likelihood-based substructure determination.
